# Developing Behavior Change Interventions for Self-Management in Chronic Illness: An Integrative Overview

**DOI:** 10.1027/1016-9040/a000330

**Published:** 2018-08-16

**Authors:** Vera Araújo-Soares, Nelli Hankonen, Justin Presseau, Angela Rodrigues, Falko F. Sniehotta

**Affiliations:** ^1^ Institute of Health & Society, Faculty of Medical Sciences, Newcastle University, Newcastle upon Tyne, UK; ^2^ School of Psychology, Faculty of Medical Sciences, Newcastle University, Newcastle upon Tyne, UK; ^3^ Faculty of Social Sciences, University of Tampere, Finland; ^4^ Clinical Epidemiology Program, Ottawa Hospital Research Institute, Ottawa, Canada; ^5^ School of Epidemiology, Public Health and Preventive Medicine, University of Ottawa, Canada; ^6^ School of Psychology, University of Ottawa, Canada; ^7^ Fuse. The UK Clinical Research Collaboration Centre for Translational Research in Public Health

**Keywords:** Behavior change, intervention development, complex interventions

## Abstract

**Abstract.** More people than ever are living longer with chronic conditions such as obesity, type 2 diabetes, and heart disease. Behavior change for effective self-management can improve health outcomes and quality of life in people living with such chronic illnesses. The science of developing behavior change interventions with impact for patients aims to optimize the reach, effectiveness, adoption, implementation, and maintenance of interventions and rigorous evaluation of outcomes and processes of behavior change. The development of new services and technologies offers opportunities to enhance the scope of delivery of interventions to support behavior change and self-management at scale. Herein, we review key contemporary approaches to intervention development, provide a critical overview, and integrate these approaches into a pragmatic, user-friendly framework to rigorously guide decision-making in behavior change intervention development. Moreover, we highlight novel emerging methods for rapid and agile intervention development. On-going progress in the science of intervention development is needed to remain in step with such new developments and to continue to leverage behavioral science’s capacity to contribute to optimizing interventions, modify behavior, and facilitate self-management in individuals living with chronic illness.

Life expectancy continues to increase worldwide, with the global average life expectancy having increased by 5 years between 2000 and 2015 (World Health Organization, 2014a). However, non-communicable conditions such as cardiovascular disease, respiratory disease, cancer, and diabetes have also increased since 2000 in every region of the world and are now the most prevalent causes of mortality and morbidity (World Health Organization, 2014a, 2014b). Chronic non-communicable conditions share behavioral risk factors such as tobacco smoking, poor diet, and physical inactivity (Lim et al., 2012). These conditions are also associated with an increased risk of undermining mental health (Moussavi et al., 2007). Multimorbidity is also prevalent and health behaviors can benefit patients by positively impacting on more than one condition (Barnett et al., 2012). Self-management is thus a complex endeavor, involving adherence to treatment, change to multiple health behaviors, and regular contact with healthcare providers (Department of Health, 2012; Schulman-Green et al., 2012).

Interventions addressing risk factors and supporting behavior change for the effective self-management of chronic conditions can make a considerable difference to health and well-being and reduce the costs of delivering health care to an aging population living longer with chronic conditions (OECD/EU, 2016). In the US, 157 million people are predicted to live with chronic conditions by 2020. Population aging raises capacity concerns for healthcare systems, in their current configurations, to cope with the increasing burden of chronic conditions (Bodenheimer, Chen, & Bennett, 2009; NHS England, 2016). There is consensus for the need for interventions to support individuals and populations by targeting the prevention and self-management of chronic disease (Boon et al., 2014) and for the key role of behavior change interventions in this process (Hardeman, Sutton, Michie, & Kinmonth, 2004).

## What Is a Health Behavior Change Intervention?

Interventions are coordinated sets of activities and techniques introduced at a given time and place to change the behavior of individuals, communities, and/or populations through a hypothesized or known mechanism (NICE, 2007, 2014). The health of populations and the individuals within them is influenced by a complex system of determinants, from individual lifestyle factor to community influences, through living, working, and social conditions (Dahlgren & Whitehead, 2006). Health behavior change interventions can be targeted at a combination of levels: policy (e.g., laws and regulation), community (e.g., neighborhoods), macro-environments (e.g., foot outlets or transport links), micro-environmental (e.g., choice architecture in shops), institutional (e.g., schools and employers), interpersonal (families and social networks), and/or intrapersonal (e.g., weight loss program or therapy) level (Araújo-Soares & Sniehotta, 2017; Hollands et al., 2017; McLeroy, Bibeau, Steckler, & Glanz, 1988).

Health behavior change interventions are usually complex (Craig et al., 2008). What makes an intervention complex is the number and complexity of its interacting components, the behaviors involved, the organizational group, and individual levels targeted and the outcomes as well as the degree of flexibility or tailoring permitted. The TIDieR checklist (Hoffmann et al., 2014) was developed to improve the completeness of reporting, and ultimately the replicability, of interventions by describing: (a) a rationale or theory describing the goals of the intervention elements, (b) the content in terms of behavior change methods (Adams, Giles, McColl, & Sniehotta, 2014; Hollands et al., 2017; Kok et al., 2016; Michie, Richardson, Johnston, Abraham, Francis, Hardeman, et al., 2013), materials, and procedures, (c) provider(s) (including qualification and training needed), (d) modes of delivery (e.g., provided face-to-face or through a digital platform) to individuals or groups (Dombrowski, O’Carroll, & Williams, 2016), (e) location and required infrastructure, (f) timing and dose, and (g) any planned mechanisms for tailoring or adaptation of the intervention to needs/features of the recipient(s). An extension of the TIDieR guideline for reporting population health and policy interventions has recently been published (Campbell et al., 2018). Interventions also often include additional components to build and sustain rapport and engagement through interpersonal styles (Hagger & Hardcastle, 2014) or features such as gamification in digital interventions (Cugelman, 2013). Health behavior change intervention development is the process of deciding the optimal combination of these features and the transparent reporting of these decisions.

## What Makes a Good Health Behavior Change Intervention?

“Primum non nocere” (eng. “first, do no harm”). The principle of non-maleficence is the single most important criterion for any health intervention (Craig et al., 2008; Michie, Atkins, & West, 2014). In addition, a good intervention should be designed for impact, should be evaluable, should not increase social inequalities, and should have a demonstrable benefit over existing interventions and services.

The impact of interventions on the health of the target audience can be illustrated through the RE-AIM (Reach, Effectiveness, Adoption, Implementation, Maintenance) model (Glasgow, Vogt, & Boles, 1999). Reach refers to the proportion of the intended target population that can actually be and is ultimately reached with an intervention; Effectiveness refers to the beneficial and unintended effect the intervention achieves on key outcomes under real-world conditions, including cost-effectiveness; Adoption refers to the uptake of the intervention by the staff, settings, and organizations; Implementation refers to the degree to which the intervention can/will be delivered consistently and with fidelity over time and setting; and Maintenance refers to the sustainability of intervention effectiveness in individuals and settings over time. To achieve this, interventions should be based on the best available evidence-based theory and direct evidence to optimize impact and to model whether and how the intervention is likely to create benefit (Bartholomew Eldredge et al., 2016; Craig et al., 2008; Wight, Wimbush, Jepson, & Doi, 2016). Optimizing RE-AIM is aided by maximizing the acceptability and feasibility of intervention procedures and materials (Lancaster, 2015). This is best achieved through the active involvement of key stakeholders in all stages, from development through to evaluation of acceptability and feasibility in initial pilot/feasibility studies as well as subsequent efficacy/effectiveness, implementation and maintenance evaluations (Craig et al., 2008; O’Brien et al., 2016).

A prerequisite of a good intervention is its “evaluability,” that is, whether its effect can be robustly evaluated. Interventions with a clear definition, elaborated logic model, and defined primary and intermediate targets are easier to evaluate, which in turn facilitate understanding if, how and for whom an intervention works, facilitating optimization and thereby contributing to the accumulation of knowledge (Leviton, Khan, Rog, Dawkins, & Cotton, 2010; Ogilvie et al., 2011; Windsor, 2015).

Good interventions should not increase social inequalities in health (Lorenc, Petticrew, Welch, & Tugwell, 2013). Health and healthy life expectancy are strongly related to socioeconomic status (OECD/EU, 2016). To avoid intervention-generated inequalities, intervention design should be sensitive to PROGRESS indicators (Place of residence, Race/ethnicity/culture/language, Occupation, Gender/sex, Religion, Education, Socioeconomic status, and Social capital (T. Evans & Brown, 2003; O’Neill et al., 2014). Intervention developers need to consider uptake, usage, and level of individual agency required to minimize the potential of generating inequalities (Adams, Mytton, White, & Monsivais, 2016).

Finally, good interventions should create incremental benefit over already existing interventions and services. Interventions have high utility if they address gaps in provision, increase the potential to be implemented and sustained, reduce costs and/or address barriers compared with previous and existing interventions. In particular, scalable interventions, that is, effective interventions which have a far reach and modest costs, address the need for solutions which have few resource and geographic barriers and can be provided to large numbers of individuals and communities (Milat, King, Bauman, & Redman, 2013). The health research landscape is not short of behavioral interventions. In light of this, a thorough environmental scan analysis is needed to identify gaps in provision to ensure that new interventions have a fair chance to make a positive contribution to health and well-being. Understanding usual care and competing interventions in a given setting enables strategic decision-making about potential incremental benefit of a new intervention. Increasingly, the boundaries of usual care are no longer physical or geographical. As interventions can take years to be developed and fully evaluated, this analysis of the health intervention market should also consider pilot studies and evaluation studies underway, for example, by analyzing trial registries and grey literature (Adams, Hillier-Brown, et al., 2016).

## The Process of Intervention Development

There is a range of frameworks that can inform the development of health behavior change interventions such as the MRC guidance for the development and evaluation of complex interventions (Craig et al., 2008), Intervention mapping (IM; Bartholomew Eldredge et al., 2016), Theory Informed Implementation Intervention (S. D. French et al., 2012), PRECEDE-PROCEDE (Green & Kreuter, 2005), the Person-Based Approach (Yardley, Morrison, Bradbury, & Muller, 2015), the 6SQuID approach in quality intervention development (Wight et al., 2016), evidence-guided co-design (O’Brien et al., 2016), the Knowledge-to-Action (KTA) cycle (Graham et al., 2006), the ORBIT model (Czajkowski et al., 2015), the Experimental Medicine Model (Sheeran, Klein, & Rothman, 2017), Multiphase optimization strategy (MOST; Collins, Murphy, & Strecher, 2007), and the Behavior Change Wheel (Michie, van Stralen, & West, 2011; see Appendix A for a summary of frameworks and their purpose). While each has a different focus and approach, they converge on a core set of key steps that include: analyzing the problem and developing an intervention objective, causal modeling, defining intervention features, developing a logic model of change, developing materials and interface, and empirical optimization followed by outcome and process evaluation and implementation. Intervention development is iterative, recursive, and cyclical rather than linear. Developers may need to go back and forth between steps to achieve the optimal intervention definition paired with most appropriate logic model of change within available resources.

Intervention development should ideally be led by an interdisciplinary Planning and Development Group representing relevant expertise (e.g., clinical care, psychology, policy, sociology, health economics, epidemiology, service design) and key stakeholders (e.g., citizens, patients, carers, healthcare professionals, deliverers, commissioners, policymakers, funders) to understand the context for intervening and to make strategic decisions that reflect scientific evidence and the preferences and views of those for whom the intervention is developed and those whose input is needed to adopt and implement the intervention (Bartholomew Eldredge et al., 2016; Witteman et al., 2017). To document the sequence of decisions involved in intervention development, workbooks can help to record intervention development steps, crucial decisions, and the process and information informing these decisions (Bartholomew Eldredge et al., 2016); Appendix B contains a comprehensive list of Key Considerations for the Reporting of Intervention Development). Next, we address each key step in detail:

### A. Analyzing the Problem and Developing an Intervention Objective

The development of a behavior change intervention rests on a foundation of a thorough analysis of the problem that the intervention developers aim to solve and a clear definition of intervention objectives. PRECEDE/PROCEED was conceived in the 1970s to guide policymakers and intervention planners in analyzing the likely costs and benefits of health programs. It consists of two main parts: PRECEDE describes an “educational diagnosis” and is an acronym for Predisposing, Reinforcing and Enabling Constructs in Educational Diagnosis and Evaluation. PROCEED refers to an “ecological diagnosis” and stands for Policy, Regulatory, and Organizational Constructs in Educational and Environmental Development (Green & Kreuter, 2005). It provides the first framework for analyzing how health and quality of life relate to behavior, physiology, and environmental factors and for the identification of predisposing, reinforcing, and enabling factors for behaviors, which can be tackled with interventions.

Many intervention development frameworks include a Needs Assessment, which involves assessing the health problem and its likely behavioral, social, and environmental causes. This initial stage involves the identification and definition of the sequence of behaviors needed to modify health outcomes thereby identifying intermediate outcomes relevant for the hypothesized mechanisms of the intervention (Bartholomew Eldredge et al., 2016), that is, “who needs to do what differently, when, where, how?” (S. D. French et al., 2012). The person-based approach to intervention development (Yardley et al., 2015) aims to ground the development of behavior change interventions in an understanding of the perspective and psychosocial context of the people who will use them. Behaviors targeted for change are embedded in a network of multiple behaviors, some of which may facilitate or conflict with each other (Presseau, Tait, Johnston, Francis, & Sniehotta, 2013). Understanding how a target health behavior fits alongside other behaviors, and the essential preparatory behaviors required, can help to identify the most viable behavioral targets for an intervention that may extend beyond the single behavioral outcome of the intervention. Target behaviors need to be defined in context and in very specific terms, ideally in terms of Target(s), Action, Context(s), Time(s) and actors (Fishbein, 1967; Francis & Presseau, 2018), including the inter-relationships between behaviors and actors. Considerations about changeability guide the prioritization and selection of target behaviors and targeted antecedents of behavior, for example, which changes are achievable based on current evidence and theory, and how much impact would such changes have for the key outcomes (Bartholomew Eldredge et al., 2016; Czajkowski et al., 2015; Sheeran et al., 2017; Wight et al., 2016).

Key stakeholders should contribute from the beginning to defining the initial problem, rather than the intervention development being a researcher-driven top-down design task. Stakeholder involvement helps to bridge between the evidence and the local context and ensures ownership, acceptability, and widespread support for the intervention essential for implementation (O’Brien et al., 2016). In some instances, intervention priorities are driven by users or patient organization. Such priorities can be robustly surfaced, for example, involving James Lind Alliance (2017) methods that bring clinicians, patients, and carers together to use a formal methodological approach to generate research priorities that are important to patients across a range of settings.

### B. Defining the Scientific Core of the Intervention

Health behavior change interventions are guided by a logic model or a theory of change that combines the intervention techniques used to target causal mechanisms into a comprehensive and testable set of assumptions (Moore et al., 2015). Three steps go hand in hand and are best described as one iterative process:(i) causal modeling of the problem, (ii) defining intervention features, and (iii) formulating a logic model of change for the intervention (Bartholomew Eldredge et al., 2016; Moore et al., 2015; Wight et al., 2016).

Decisions need to be made on method(s) and mode(s) of delivery, behavior change technique(s), provider(s), location(s), timing, dose, personalization and hypothesized causal mechanisms to optimize reach, (cost-) effectiveness, adoption, implementation, and maintenance. These design decisions should be recorded and made explicit to clarify the contribution that all new interventions make to previous evidence. The process should be led by a participatory planning group representing stakeholders such as users and commissioners of the intervention and the research team to iteratively build a hypothesis of change and make design decisions based on scientific evidence and the needs of the target audience. This ensures the relevance of the developed solution and creates co-ownership as a result of coproduction.

#### (i) Causal Modeling

The identification of causal and contextual factors affecting self-management behaviors is a key step in intervention development. Behavior is the result of a complex ecologic system of influences which range from proximal individual, cognitive, and emotional factors to social and community influence up to more distal factors such as care delivery systems (e.g., access to specialist medical care), living and working conditions (employment, environment, education, and housing), and socioeconomic, cultural, and environmental conditions (e.g., legislation; Dahlgren & Whitehead, 2006). Modifiable factors that have a strong relationship to the target behavior are potential targets for interventions (Michie, van Stralen, et al., 2011; Wight et al., 2016).

Behavior change approaches tend to operate on the assumption that interventions affect behavior by modifying social, environmental, and/or cognitive predictors of the target behavior. Interventions are then thought to operate through a sequential causal model beginning from predictors of behavior, to behavior, to physiological changes and eventually leading to health outcome(s) (Hardeman et al., 2005). IM (Bartholomew Eldredge et al., 2016) proposes to work backward from the targeted health problems (and that impact on quality of life), to the behavior and environmental factors that shape these health problems, and finally to the predictors of the causal behavioral and environmental risk factors. Predictors are rated by relevance and changeability to determine their priority for inclusion in the intervention (Bartholomew Eldredge et al., 2016; Yardley et al., 2015).

Literature reviews are recommended to synthesize evidence of the causes and predictors of the target behavior (Bartholomew Eldredge et al., 2016; Craig et al., 2008), ideally, with systematic searches (Craig et al 2008). In reviewing existing evidence, tensions between strength and rigor and applicability of evidence can occur. Decisions about evidence reviews should be strategically driven to address key uncertainties. While usually systematic reviews of studies with low risk of bias are preferable, the most relevant evidence informing an intervention might be supplemented by grey literature such as local government reports or hospital records (Adams, Hillier-Brown, et al., 2016; O’Brien et al., 2016; Rodrigues, Sniehotta, Birch-Machin, Olivier, & Araujo-Soares, 2017). Reviews may highlight the degree to which results are likely to be transferable to the present context but often additional empirical research is needed to identify the most important predictors and to test their sensitivity to contextual features of communities, services, or geographies.

Theory has a central role in this process. Intervention development is often based on operationalizing the principles from a single theory and selecting intervention techniques with the potential to modify the theoretical predictors of behavior. This approach can be useful when there is insufficient resource to consider collecting further empirical data and given the inherently evidenced-based nature of a theory, in that it has been successfully applied to different behaviors and/or in different contexts (D. P. French, Darker, Eves, & Sniehotta, 2013). However, this approach is limited when the observed prospective relationships considered for the selection of intermediate intervention targets are not strong enough for interventions changing behavioral predictors to achieve changes in behavior (Sniehotta, Presseau, & Araújo-Soares, 2014).

When no appropriate theory can be identified, or when more than one may seem relevant, intervention developers can use the Theoretical Domains Framework (TDF) to organize evidence about key barriers and enablers and link back to relevant theories (Francis, O’Connor, & Curran, 2012; Heslehurst et al., 2014). The TDF is a simple tool developed through review and consensus methods to describe the most common explanatory constructs in behavioral theories organized into 14 domains: knowledge, skills, social influences, memory, attention and decision processes, social/professional role and identity, reinforcement, beliefs about capabilities, beliefs about consequences, optimism, intention, goals, behavioral regulation, emotion, environmental context and resources (Cane, O’Connor, & Michie, 2012; Michie et al., 2005). The TDF can be used to inform both qualitative and quantitative studies with the aim to understand key predictors of behavior and to identify the most relevant theoretical approach (Beenstock et al., 2012; Laine, Araújo-Soares, Haukkala, & Hankonen, 2017; Presseau, Schwalm, et al., 2017).

Additional empirical studies can increase understanding of the key influences of the behavior in the target group. For example, a survey identifying the most important correlates of physical activity behavior and intention could help in selecting the key barriers and enablers to target with an intervention (Hankonen, Heino, Kujala, et al., 2017; Presseau, Schwalm, et al., 2017; Sniehotta, Schwarzer, Scholz, & Schüz, 2005). Qualitative interviews or *n*-of-1 studies can provide an individualized assessment of barriers and needs (McDonald et al., 2017; Rodrigues, Sniehotta, Birch-Machin, & Araujo-Soares, 2017; Yardley et al., 2015). A key weakness of approaches based on correlation is the lack of causation and the problem of attenuation, that is, large changes in predictors are needed to achieve modest changes in behavior (Sniehotta, 2009).

**Figure 1:**
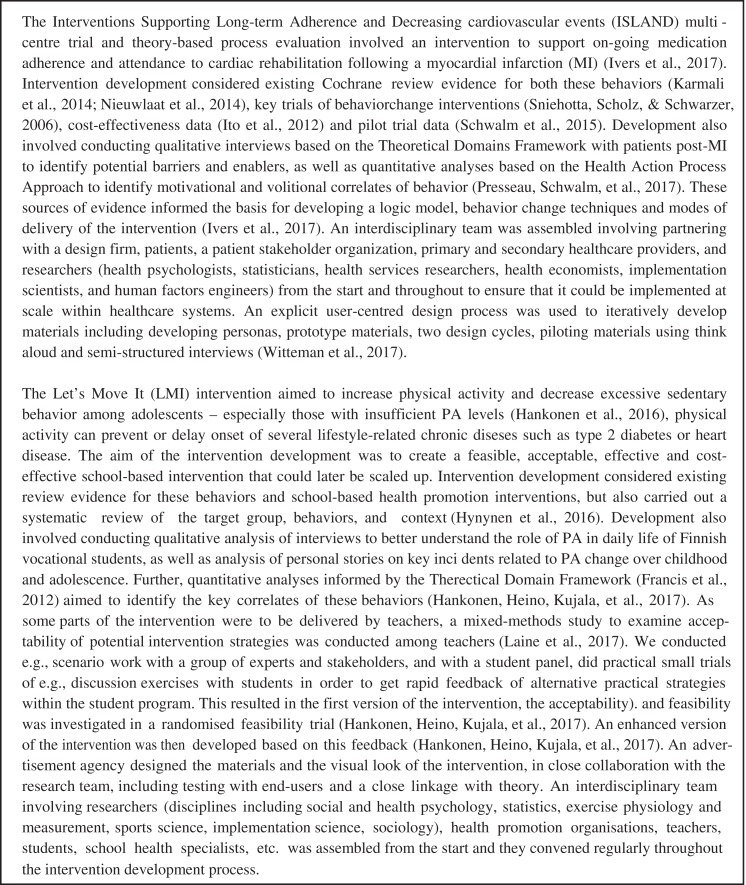
Intervention development examples.

Where multiple behaviors are targeted, a process of testing multiple theories across multiple behaviors can be used to identify the most consistently predictive constructs within their theories across behaviors, then theorize and test how such theories and their constructs can be combined, for example, into a dual process model (Presseau, Johnston, et al., 2014) to inform a logic model (Presseau, Hawthorne, et al., 2014). This approach combines the strength of preexisting theory (and its tested mediating and moderating mechanisms) with the empirical comparison of theory across behaviors to facilitate the selection of behavior(s) and theory upon which to further develop the intervention. Theory is used to address uncertainties and may include theoretical ideas that are not directly related to behavior, for example, theories of persuasion (Petty & Cacioppo, 1986) or of symptom recognition (Petersen, van den Berg, Janssens, & van den Bergh, 2011). Figure 1 provides two examples of intervention development.

#### (ii) Defining Intervention Features

Intervention techniques (e.g., to change behavior, cognitions, perceptions, or environmental variables) are selected based on evidence of their effectiveness in changing the identified causal and contextual factors influencing the target behavior. Intervention development approaches differ in how they approach the analysis of causal factors focussing on intervention targets or techniques (Michie et al., 2014; Sheeran et al., 2017; Webb, Michie, & Sniehotta, 2010). Target-based approaches identify modifiable predictors of behavior, whereas technique-based approaches focus on intervention techniques themselves and contextual modifications which directly influence behavior (Webb et al., 2010).

As highlighted in the knowledge creation funnel within the KTA cycle (Graham et al., 2006), use of review evidence sets the foundation and prevents repeating previously unsuccessful behavior change techniques or withholding intervention strategies with demonstrated effectiveness in changing behavior. In some cases, evidence synthesis may identify that a suitable intervention already exists that could be retrofitted (i.e., transformed for use in a novel context and or in a novel population) rather than re-invented. But systematic reviews of randomised controlled trials (RCTs) of interventions with similar aims do not always provide sufficient answers. For example, in the development of the “Let’s Move It” intervention to change physical activity and sedentary behaviors in vocational school, a systematic review (Hynynen et al., 2016) informed the designers about what works in getting older adolescents more active, but it was not sufficient. A range of other relevant sources of evidence contributed to its development including existing evidence regarding the setting (school-based health promotion), evidence about the target behavior using a range of methods and research on similar interventions in other age groups and populations contributed to inform the intervention design.

Different levels of evidence answer different questions. While systematic reviews of RCTs of behavior change interventions provide the strongest evidence for effectiveness, they often say little about reach, adoption, and implementation outside of a research study or about longer-term maintenance (Dombrowski et al., 2012). Likewise evidence from rigorous studies conducted in very different settings or in communities with different features may be applicable to the local needs when retrofitted. Evidence synthesis should be strategic and sequential, developing an iterative understanding of how to optimize the intervention (Michie et al., 2014). Where previous health behavior change interventions had heterogeneous effects, it is often possible to code behavior change techniques and other intervention features such as modes of delivery (Abraham & Michie, 2008; Adams et al., 2014; Kok et al., 2016; Michie, Ashford, et al., 2011; Michie, Richardson, Johnston, Abraham, Francis, & Hardeman, 2013) and to explore whether such features are associated with intervention effectiveness (Dombrowski et al., 2012). Such an intervention features review-based approach begins by identifying intervention techniques and other TIDIER features (Hoffmann et al., 2014) of interventions for a given health behavior in a systematic review of trials. TIDIER features, including behavior change techniques and other intervention techniques can then be coded within interventions in the review to test which techniques and combinations of these are associated with greater effectiveness in other settings. Even though trials of interventions make causal statements of effectiveness, the evaluation of intervention techniques within the review is correlational and should be treated with due care. Nevertheless, this approach can help to combine evidence of intervention strategies that have been found to be effective in other settings and/or using theory to inform the selection of intervention techniques.

In addition to review-based identification of effective intervention features, some approaches promote an experimental method for intervention development to establish causal evidence for the hypothesized change by identifying the potential modifiable causal factors and assessing whether changes in the target behavior occur as a result of manipulating the predictive factor(s) (Sheeran et al., 2017). The emphasis is on understanding the mechanisms of change and using experimental designs to robustly clarify how to change these and integrating this knowledge into applied research. Environmental interventions targeting point-of-choice decisions such as stairs versus escalator use (Ryan, Lyon, Webb, Eves, & Ryan, 2011) and on-the-spot opportunities to register for organ donation (Li et al., 2017), nudges (Hollands et al., 2013; Marteau, Ogilvie, Roland, Suhrcke, & Kelly, 2011) or point of sale decisions (Dolan et al., 2012) are more likely to be informed by experimental than by correlational considerations.

Some intervention techniques may be effective when tested in an RCT but not widely acceptable by facilitators or target audience alike, while other intervention techniques might be highly acceptable but show smaller effect sizes. Acceptability can be defined as a “multi-faceted construct that reflects the extent to which people delivering or receiving a healthcare intervention consider it to be appropriate, based on anticipated or experienced cognitive and emotional responses to the intervention” (Sekhon, Cartwright, & Francis, 2017, p. 4). Engaging stakeholders in the development process from early on will increase the potential for acceptability. Intervention principles that are theoretically sound and in line with good evidence, might still not be seen as acceptable without adaptation to context and audience. For example, some might not be willing to engage in planning interventions unless key modifications are implemented to increase acceptability and feasibility (Witteman et al., 2017). Anticipated acceptability of candidate features can be empirically examined to inform decisions, for example, teachers’ views on potential strategies to reduce student sitting in schools was examined using a mixed-methods approach (Laine et al., 2017). This example also illustrates that in addition to the main target group (students), the environmental agents or “providers” (teachers) that deliver the intervention are also the target of a “secondary” intervention, hence, their views and behaviors should also be understood. In implementation science the environmental agents are the target of the intervention.

#### (iii) Developing a Logic Model of Change

The MRC framework for the development and evaluation of complex interventions highlights that interventions should be theory-based (Craig et al., 2008). A common misconception is equating “theory” with “hypothesis.” A scientific theory has been empirically demonstrated to explain behavior. If, while designing an intervention, the team concludes that there is a need to target a combination of constructs from different theories that have never been tested together, what will actually happen is that a specific scientific hypothesis (that can lead to a new theory if successful) is being tested, not a theory.

It is useful to create a program’s scientific hypothesis in terms of the evidence-based mechanisms associated with behavior and behavior change. In contrast to formal scientific theories, program theories are practical, concrete working models and hypotheses of interventions, and are specific to each program or intervention. They (1) specify the intervention components, the intervention’s expected outcomes, and the methods for assessing those outcomes, often in the form of a logic model, and (2) offer an intervention’s “hypotheses” (the rationale and assumptions about mechanisms that link processes and inputs to (both intended and unintended) outcomes, as well as conditions/context necessary for effectiveness; Davidoff, Dixon-Woods, Leviton, & Michie, 2015).

This hypothesis of change may be based on or informed by scientific theories, but the main requirement is to formalize the hypothesized causal assumptions, detail the planned implementation and theorized mechanisms of impact within a set of relevant contexts (Craig et al., 2008). Theory can also identify specific issues that create barriers to intervention success (e.g., competing goals in time-limited GP sessions; Presseau, Sniehotta, Francis, & Campbell, 2009). Rather than using a single theory to guide intervention development, it is often sensible to use theory to address the uncertainties in the process and to create a map of assumptions/hypothesis linking theories and evidence.

According to UK MRC Guidance, modeling an intervention before evaluation provides the insights that are key to informing the design of both the intervention and its evaluation. Modeling may take the form of a pretrial economic evaluation testing if the set of assumptions used to develop the interventions are sufficient to provide a good chance of successful impact. Mapping links between outcomes, determinants, change objectives, and intervention techniques reflect this process of creating the logic of intervention (Bartholomew Eldredge et al., 2016). For example, in a school-based intervention to prevent obesity, performance objectives (e.g., Communicate healthy behavior messages to parents and seek their support) are mapped against personal (e.g., self-efficacy) and external, environmental predictors (e.g., family support), and thus created actionable change objectives (e.g., confidence to seek parental support and social reinforcement from parents/family for interest in healthy lifestyles. These change objectives become the target of intervention techniques (Lloyd, Logan, Greaves, & Wyatt, 2011).

This process should also involve the explicit elaboration of a “dark” logic model, that is, a careful elaboration of potential pathways through which the intervention may lead to negative or harmful consequences (Bonell, Jamal, Melendez-Torres, & Cummins, 2014). This extends beyond identifying potential harms by clearly outlining the mechanisms through which such harms may take place.

The Behavior Change Wheel (Michie, van Stralen, et al., 2011) is a particularly useful recent tool to integrate theory and evidence and to bring together stakeholders in making intervention design decisions. It is a meta-model of the intervention development process based on a comprehensive review and synthesis of existing methodological and theoretical approaches from various disciplines. The Behavior Change Wheel links policy categories (guidelines, environmental/social planning, communication/marketing, fiscal measures, regulation, service provision and legislation) with intervention functions (restrictions, education, persuasion, incentivization, coercion, training, enablement, modeling, and environmental restructuring) and commonly theorized sources of behavior; Capability (physical and psychological), Opportunity (social and physical) and Motivation (automatic and reflective), known as the COM-B model (Michie, van Stralen, et al., 2011).

### C. Development of Material and Interface

Design decisions about the look and feel of an intervention can promote their sustained use and are thus highly dependent on the mode of deliver, target audience and behavior. In a digital intervention, the graphics used, decisions about gamification and devices used to deploy the intervention influence the overall success of a behavior change intervention. This calls for multidisciplinary work to incorporate theories and methods from other disciplines. Health behavior change theories are not sufficient for informing all decisions about the design of an intervention, and other disciplines have a key role in optimizing design decisions. The use of community-based participatory research (Teufel-Shone, Siyuja, Watahomigie, & Irwin, 2006) such as consensus conferences (Berry, Chan, Bell, & Walker, 2012) or co-design workshops (O’Brien et al., 2016) and user-centered design (Cafazzo, Casselman, Hamming, Katzman, & Palmert, 2012) help to make the intervention attractive, clear and relevant to the user.

Producing final program materials such as posters and videos may involve creative consultants, artists or graphic designers. IM suggests writing design documents to guide the creation and reviewing of the materials: They can help in ensuring that behavioral science insights and intervention strategies are adequately transferred into actual material production.

### D. Empirical Optimization

Once the intervention program is designed and materials developed into a ‘beta’ version, there is the need for refinement and optimization. Building in time for this extra step will increase future acceptability and feasibility of the intervention. There are rigorous methods that can be used to get extra information to proceed with empirical optimization/refinement of the intervention prior to wider scale evaluation, such as the Multiphase Optimization Strategy (MOST). Qualitative and/or quantitative methods can facilitate optimization/refinement.

MOST is a framework for robust empirical optimization and evaluation of behavior change interventions (Collins et al., 2007; Collins, Nahum-Shani, & Almirall, 2014). MOST proposes three phases: preparation (i.e., develop theoretical model and highlight uncertainties about most effective intervention features), optimization (i.e., component selection using empirical testing), and evaluation (i.e., definitive RCT). At the optimization phase intervention developers gather empirical information on each intervention feature by conducting a randomized experiment (e.g., factorial design, fractional factorial design, SMART designs). The results from this formal testing inform decision-making process in terms of feature selection and formation of the optimized intervention. The framework proposes an iterative process stating that if an optimized intervention is shown to be effective through a formal test, it can be made available to the public. The key element in MOST is the processes by which a multicomponent behavior change intervention and its components are optimized before a definitive trial or potentially while the intervention is in use (e.g., optimization of an existing app).

Qualitative methods provide a complementary approach to support the development and refinement of an initially drafted intervention. Developers should aim to understand and incorporate the perspectives of those who will use the intervention by undertaking iterative qualitative research. This is important for digital interventions (Baek, Cagiltay, Boling, & Frick, 2008) but also for traditional methods of delivery. An example on how this can be translated in practice is by eliciting and analyzing service users’ reactions to the intervention and its elements. It might also be important to conduct consultation with topic experts (e.g., computer scientists) and other stakeholders (e.g., healthcare practitioners) of the intervention to accommodate their views and expertise (Presseau, Mutsaers, et al., 2017; Rodrigues, Sniehotta, Birch-Machin, Olivier, et al., 2017). This can be achieved using research methods such as focus groups, individual semi-structured interviews coupled with a think-aloud process. Mixed methods can also be used to refine an intervention coupling both qualitative with quantitative forms of collecting information that can inform refinement.

### E. Evaluating the Intervention

Developing interventions that test explicit hypotheses could allow for synergy between knowledge generated via the implementation and evaluation of interventions and theories, allowing for their test and evolution. In the pilot and feasibility stage the feasibility and acceptability of the intervention and evaluation procedures is tested and if needed optimized and additional information needed to design the evaluation is collected (Eldridge et al., 2016; Lancaster, 2015). Once a viable intervention and evaluation protocol has been achieved, a full-scale evaluation of whether the intervention has its intended effects on the main outcome should take place assuming resources are available to do so.

The study design should be chosen based on what is fit for purpose – based on question, circumstances, and specific characteristics of the study (e.g., expected effect size and likelihood of biases). Considering the range of experimental and non-experimental approaches should lead to more appropriate methodological choices (Shadish, Cook, & Campbell, 2002). UK MRC guidance strongly encourages considering randomization, due to it being the most robust method of preventing selection bias (i.e., intervention recipients systematically differing from those who do not). In case a conventional individually-randomized parallel group design is not appropriate, evaluators should consider other experimental designs, for example, cluster-randomized trials, stepped wedge designs (Li et al., 2017), preference trials and randomized consent designs, or *n*-of-1 designs (Craig et al., 2008; Shadish et al., 2002). Even when an experimental approach may not be feasible, for example, the intervention is irreversible, robust nonexperimental alternatives should be considered. In any case, intervention evaluators should be conscious of the need to avoid underpowered trials to prevent producing research waste (Ioannidis et al., 2014).

### F. Process Evaluation

In addition to a formal outcome evaluation, an important part of intervention development and evaluation involves understanding how and for whom an intervention works or does not. Process evaluation is key to explore the functioning of a complex intervention and it involves examining fidelity, mechanisms of impact, and contextual factors (Moore et al., 2015). A process evaluation can involve the use of various qualitative and/or quantitative methods to increase understanding of outcomes, how these are achieved and how can interventions be improved (Moore et al., 2015). For instance, a process evaluation can include self-completed questionnaires (E. H. Evans et al., 2015), semi-structured interviews (Sainsbury et al., 2017), data-driven interviews (Leslie et al., 2016), and non-participant observations to understand the functioning of the different features of an intervention (Hardeman et al., 2008). It should be noted that process evaluation can be conducted at various stages of intervention development and evaluation, serving a different function in each: in the feasibility and pilot study phase it may, for example, shed light on intermediate processes and acceptability of implementation procedures (Hankonen, Heino, Hynynen, et al., 2017), in the effectiveness evaluation trial, fidelity, impact mechanisms and context (Presseau et al., 2016), and finally in the post-evaluation implementation, its function may be to investigate the routine uptake or normalization into new context (May & Finch, 2009; Moore et al., 2015). For example, in the feasibility study of the “Let’s Move It” intervention to promote physical activity in vocational school youth, the identification of activities most and the least frequently taken up by the participants enabled an improvement or removal and replacement of such suboptimal program components (Hankonen, Heino, Hynynen, et al., 2017).

### G. Implementation: Real-World Application

Once a health behavior change intervention is evaluated and demonstrated to be effective, this evaluation contributes to the wider evidence in favor of the intervention. As replicated evidence mounts and is synthesized in favour of the intervention, there can be greater confidence in promoting its implementation and routine use as part of a new standard of care in health services, community services, schools, the workplace and/or online (Peters, Adam, Alonge, Agyepong, & Tran, 2013). Demonstrating that an intervention is effective does not guarantee that it will be adopted or implemented beyond the scope of the project that developed and evaluated it. As suggested within RE-AIM, real-world implementation issues should be integrated as a key consideration at each stage of an intervention’s development and evaluation process. Intervention co-creation provides some ownership to those involved with its implementation but does not guarantee that others will use it. The field of Implementation Science has emerged to robustly develop and evaluate interventions to support real-world implementation process itself. The “actors” whose behavior is targeted thus shifts from patients and citizens, to those who deliver the intervention in routine settings (e.g., doctors, nurses, teachers), and the same rigorous process of intervention design advocated above for patient/citizen-focused interventions should form the basis of an implementation intervention, including development, piloting and evaluation. Just as mere information provision is unlikely to support someone to quit smoking or eat more healthily, so too is the provision of information to a healthcare provider about an effective health behavior change intervention unlikely to be sufficient to change routine practice. Instead, change in healthcare provider behavior should be assessed and informed by behavior change theory qualitatively, quantitatively, determinants reviewed, pilot testing, and robust randomized evaluation conducted. Indeed, Cochrane reviews of strategies for supporting healthcare professional behavior exist (e.g., Ivers et al., 2012), and there is a movement toward clarifying behavior change techniques targeting change in healthcare provider behaviors alongside those focused on patients (Presseau et al., 2015). Such implementation research is best achieved in collaboration with those with the infrastructure within which to implement the intervention (e.g., health services, schools). There remains much opportunity to apply principles of behavior change intervention development and evaluation to changing the behavior of those who deliver interventions routinely.

## Conclusion: Reflections and Challenges

Methods for behavior change intervention development have progressed considerably over the last four decades and made a significant contribution to the translation of health behavior science into public health and health care. Guidance for the outcome and process evaluation of complex interventions has increased both the quality of interventions as well as their reporting (Hoffmann et al., 2014). Moving away from an academically dominated approach toward a multidisciplinary process with meaningful involvement of stakeholders and users working toward codesign and joint ownership while maintaining commitment to evidence-based practice and scientific theory, has considerably increased the potential for impact in the real world. This further underscores that reach, implementation, adoption, and maintenance – not just effectiveness – must be optimized to create maximal impact. Intervening is increasingly seen from a complex systems perspective with a view to modifying the behavioral as well as the wider social and environmental determinants of behavior and recent developments reflect this emphasis on environmental interventions and context (Aunger & Curtis, 2016; Dolan et al., 2012; Hollands et al., 2017).

Policy and practice partners often require solutions in a timely fashion and at limited budgets. Scientific methods are usually conceived to reach optimal solutions but impact might depend on creating the optimal solution in a given context of time and budget. Increasing chances of acceptability and feasibility by involving key stakeholders from the start, we can design interventions that have the highest likelihood of delivery to time and budget. These stakeholders ideally include policymakers and other agents who are gatekeepers to long-term implementation and dissemination. By partnering early and over the long term the seeds for incremental evaluation will be sow. This will increase flexibility and allow for immediate response to identified needs while also contributing to science over the longer term. Hence, involving them early on enables longsighted planning for real-world impact.

Intervention development frequently involves a systematic review, extensive patient and public involvement and additional original mixed method research before conducting a feasibility study and subsequently for a definitive study evaluating the effectiveness. While defensibly robust, this best practice approach can be time consuming, which may be appropriate in many settings. However, in domains characterized by very rapid innovation cycles, such as mobile phone apps for public health, more efficient approaches are needed and can be considered. One option rarely raised in this literature is the option not to develop an intervention but to adapt or retrofit an existing one. Such an approach is sensible where evidence synthesis or a scoping review suggests that an existing intervention has a good evidence base. An example of an adapted intervention is the “Waste the Waist,” (Gillison et al., 2012) which was based on an intervention used in Australia (Absetz et al., 2007; Laatikainen et al., 2007).

We suggest that intervention developers should avoid following formal methods in a linear “cookbook” fashion. Instead, we advocate for transparency of reporting of strategic decisions inspired by an iterative value of information approach where at each stage of the development the opportunity costs for conducting additional research or seeking further evidence is weighted against the likely improvement to the interventions resulting from it – informed by a strong multidisciplinary conceptual model. This allows some flexibility and adjusts the process to the available time and resource. It is important to highlight which design decisions are based on evidence but also to acknowledge those decisions made in the process of intervention development that could not be based on available evidence.

Finally, it is possible to use methods of empirical optimisation such as MOST (Collins et al., 2007), sequential multiple assignment randomized trial (SMART; Cheung, Chakraborty, & Davidson, 2015) or built in *n*-of-1 trials (McDonald et al., 2017) to empirically optimize interventions while being used, a possibility that benefits particularly from digital intervention platforms. Developing real-world interventions is an opportunity to create impact from behavioral science and to contribute to addressing some of the most pressing issues of our time.

## Appendix A

### Intervention Development and Evaluation Frameworks and Purpose

**Table:**

Frameworks	Purpose
MRC Framework for the Development of Complex Interventions (Craig et al., 2008)	To provide guidance on the process of development, evaluation and implementation of a target intervention.
Intervention Mapping (Bartholomew Eldredge et al., 2016)	To describe the iterative path (six phases) for designing, implementing and evaluating an intervention.
Steps for developing a theory-informed implementation intervention (S. D. French et al., 2012)	To support the development of an intervention designed to change clinical behaviour based on a theoretical framework.
PRECEDE-PROCEEDE (Green & Kreuter, 2005)	The model aims to explain health-related behaviours and environments, and to design and evaluate the intervention.
The Behaviour Change Wheel (Michie, Atkins, & West, 2014)	This tool details how to design and select interventions according to a behaviour analysis, mechanisms of action, and the interventions required to change those mechanisms. This tool is also used to link influences on behaviour to potential intervention functions and policy categories.
The Person-Based Approach to Intervention Development (Yardley, Morrison, Bradbury & Muller, 2015)	To design interventions based on rigorous, in-depth understanding of the psychosocial context of users, and derived from iterative in-depth qualitative research.
6SQuID: 6 steps in quality intervention development (Wight, Wimbush, Jepson, & Doi, 2016)	To provide a pragmatic and systematic six-step guide to intervention development, maximising its likely effectiveness.
Evidence-guided co-design (O’Brien et al., 2016)	To describe a systematic, sequential approach to integrate scientific evidence, expert knowledge, and stakeholder involvement in the co-design and development of an intervention.
Knowledge-to-Action (KTA) cycle (Graham et al., 2006)	A conceptual framework to integrate the roles of knowledge creation and knowledge application, contributing to sustainable, evidence-based interventions.
ORBIT model (Czajkowski et al., 2015)	To provide guidance on the process of treatment development by suggesting the use of a progressive, transdisciplinary framework to facilitate the translation of basic behavioural science findings to clinical application.
EM Model (Sheeran, Klein, & Rothman, 2017)	To detail the process involved in designing interventions to gain more cumulative science of health behaviour change.
Multiphase optimization strategy (MOST; Collins, Murphy, & Strecher, 2007)	To provide a guide to the optimization and evaluation of multicomponent behavioural interventions.
Social Marketing (e.g., Lefebvre, 2011)	The systematic application of marketing concepts and techniques to achieve behaviour change.

## Appendix B

### Key Considerations for the Reporting of Intervention Development

Preparatory work: Describe the team and planned development process

a. Describe the expertise of the core team and advisory stakeholder team involved in development/design process (in different phases): multi-disciplinarity, prior experience
b. Describe time used (and available) for intervention development process (e.g. length of design period, frequency of design meetings, etc.)
c. Describe other resources available
d. Describe possible funder/commissioner demands/limitations/requests for the intervention or the development process (e.g. future use, use of technology, limited financial resources, quick timeline for development)
e. Describe original general aims and intended use/scalability of the future intervention

Step 1: Analyse the problem and develop an intervention objective

a. Describe how the planning group worked to define the health problem, health behaviors, target health behaviors
b. Describe potential market analysis, segmentation, and possible subsequent resulting decisions
c. Describe the decision process leading to prioritisation and selection of target group(s) and behavior change targets
d. Describe how preparatory behaviors and networks of other behaviors were identified and prioritised

Step 2: Define the scientific core of the intervention

(i) Understand causal/contextual factors (Causal Modelling)
  a. Describe formal (behavioral) theories used in understanding the predictors of the target health behavior
  b. Describe how key uncertainties were identified to select aim of evidence synthesis
  c. Describe literature search and review process
  d. Describe the rationale/aims and the process of (possible) original empirical research
  e. Describe rating of influencing factors (psychological, social, predictors/mechanisms) for changeability and relevance
(ii) Develop a logic/theoretical model
  a. Describe the process of developing the logic model (if possible, include early and later versions of the logic model)
  b. Describe key explicit criteria (e.g. acceptability, cost-effectiveness) in making decisions for logic model
  c. Describe whether and which other similar existing interventions were used in developing the logic model, or whether an existing intervention was used as core basis and retrofitted to account for new context
  d. Describe key uncertainties left in the causal chain or logic model and the possible “weak links” the development team thinks there may remain
  e. Assess evaluability potential of such an intervention
  f. Develop a dark logic model that describes considerations made around potential unintended consequences and steps made to avoid it
(iii) Define intervention features
  a. Describe decision processes (including considered alternative options) leading to decisions about
    i. program components/activities
    ii. intermediate targets
    iii. behavior change techniques or methods to target predictors/mechanisms e.g. to what extent various combinations of BCTs were explicitly considered and left out
    iv. dose/intensity/frequency/duration of intervention
    v. delivery channel(s)
    vi. providers (expertise/background/training)
    vii. location/infrastructure
  b. Describe whether and how anticipated acceptability of intervention among target participants and/or providers and/or commissioners was investigated
  c. Describe the decision processes related to room for local adaptation and necessity of fidelity for various components

Step 3: Design/Develop intervention materials

a. Describe how protocol was written
b. Describe key principles in designing materials (e.g. design documents)
c. Describe how stakeholder input was obtained for key decisions (e.g., scenario-based work)
d. Describe whether and how small-scale pre-testing of intervention components (e.g. group exercises, key messages) was conducted, to make decisions about program content
e. Describe decisions leading to personalization and tailoring (how and why)
f. Describe the process of developing procedures to ensure fidelity

Step 4: Conduct an empirical optimization

a. Describe key (research) questions for empirical optimisation
b. Describe empirical design used in testing the intervention (or its components), including data collection methods, sample, etc.
c. Describe data analysis methods
d. Describe whether and how qualitative and quantitative methods were mixed
e. Describe how judgments and optimization decisions were made based on empirical testing

Step 5: Design and undertake intervention evaluation

a. Describe the plan for evaluation of effectiveness
b. Describe rationale (e.g. resources available, funder interests) leading to decisions regarding evaluation
c. Describe the plan for evaluating processes
d. Describe the intended use of information gained (e.g. for potential adaptations)

Step 6: Design implementation and undertake implementation evaluation

a. Describe how decisions related to implementation (specific plans on how the intervention will be used in routine practice) were done, e.g., was the implementation informed by a theoretical framework or a model
b. Describe the implementation intervention development process
c. Describe reach and allowed adaptations
d. Describe the plan for evaluation of implementation
e. Describe rationale (e.g. resources available, funder interests) leading to decisions regarding evaluation
f. Describe the plan for evaluating processes of implementation
g. Describe the intended use of information gained (e.g. for potential adaptations)
